# Marine Antimicrobial Peptide TP4 Exerts Anticancer Effects on Human Synovial Sarcoma Cells via Calcium Overload, Reactive Oxygen Species Production and Mitochondrial Hyperpolarization

**DOI:** 10.3390/md19020093

**Published:** 2021-02-05

**Authors:** Bor-Chyuan Su, Giun-Yi Hung, Yun-Chieh Tu, Wei-Chen Yeh, Meng-Chieh Lin, Jyh-Yih Chen

**Affiliations:** 1 Department of Anatomy and Cell Biology, School of Medicine, College of Medicine, Taipei Medical University, Taipei 110, Taiwan; subc8265@tmu.edu.tw; 2 Division of Pediatric Hematology and Oncology, Department of Pediatrics, Taipei Veterans General Hospital, Taipei 11217, Taiwan; gyhung@vghtpe.gov.tw; 3 Faculty of Medicine, School of Medicine, National Yang Ming Chiao Tung University, Taipei 11221, Taiwan; 4 School of Medicine, College of Medicine, Taipei Medical University, Taipei 110, Taiwan; b101108090@tmu.edu.tw (Y.-C.T.); b101108087@tmu.edu.tw (W.-C.Y.); 5 School of Medical Laboratory Science and Biotechnology, College of Medical Science and Technology, Taipei Medical University, Taipei 110, Taiwan; b614108011@tmu.edu.tw; 6 Marine Research Station, Institute of Cellular and Organismic Biology, Academia Sinica, Jiaushi, Ilan 262, Taiwan; 7 The iEGG and Animal Biotechnology Center, National Chung Hsing University, Taichung City 402, Taiwan

**Keywords:** TP4, marine antimicrobial peptide, human synovial sarcoma, calcium overload, mitochondria

## Abstract

Synovial sarcoma is a rare but aggressive soft-tissue sarcoma associated with translocation t(X;18). Metastasis occurs in approximately 50% of all patients, and curative outcomes are difficult to achieve in this group. Since the efficacies of current therapeutic approaches for metastatic synovial sarcoma remain limited, new therapeutic agents are urgently needed. Tilapia piscidin 4 (TP4), a marine antimicrobial peptide, is known to exhibit multiple biological functions, including anti-bacterial, wound-healing, immunomodulatory, and anticancer activities. In the present study, we assessed the anticancer activity of TP4 in human synovial sarcoma cells and determined the underlying mechanisms. We first demonstrated that TP4 can induce necrotic cell death in human synovial sarcoma AsKa-SS and SW982 cells lines. In addition, we saw that TP4 initiates reactive oxygen species (ROS) production and downregulates antioxidant proteins, such as uncoupling protein-2, superoxide dismutase (SOD)-1, and SOD-2. Moreover, TP4-induced mitochondrial hyperpolarization is followed by elevation of mitochondrial ROS. Calcium overload is also triggered by TP4, and cell death can be attenuated by a necrosis inhibitor, ROS scavenger or calcium chelator. In our experiments, TP4 displayed strong anticancer activity in human synovial sarcoma cells by disrupting oxidative status, promoting mitochondrial hyperpolarization and causing calcium overload.

## 1. Introduction

Synovial sarcoma is a type of soft tissue sarcoma that can occur anywhere in the body [1]. It is a rare disease, with an estimated annual incidence of 1.1 to 1.7 cases per million people in the United States, Switzerland, the EU and Taiwan [1,2]. Although it is rare, synovial sarcoma is most common in adolescents and young adults, with a median age at diagnosis of 35 years; notably, 17.6% of synovial sarcomas are diagnosed in patients under 20 years of age [3]. The precise etiology of synovial sarcoma remains unclear [4,5], however, a characteristic *SYT-SSX* fusion gene, which results from a t(X;18) translocation and represents a fusion of *SYT* (at 18q11) with *SSX1*, *SSX2* or *SSX4* (at Xp11), can be detected in more than 90% of synovial sarcomas [6]. The prognostic implications of *SYT-SSX1* and *SYT-SSX2* have been reported in two large studies with contradictory results [6,7]. Moreover, other prognostic factors of adverse patient outcomes include monophasic and poorly differentiated subtypes [8], male sex [9], advanced age at diagnosis [10], size ≥ 5 cm [11], non-limb site [11], deep-seated tumors [12], and positive surgical margin.

The survival rate of synovial sarcoma decreases with age. In the United States, the 5-year relative survival rate is 77–92% for those younger than 20 years, 55–68% for those between 20 and 59 years, and 38–52% for those 60 years or older [3]. The treatment of synovial sarcoma depends on the primary site, size, and stage of the tumor. For localized disease, wide surgical excision is the mainstay treatment. However, metastasis occurs in approximately 50% of cases, and curative outcomes are difficult to achieve in this group of patients [13,14]. For locally advanced or metastatic disease, systemic chemotherapy regimens with anthracyclines and/or ifosfamide, pazopanib, or trabectedin have shown some efficacy when used as different lines of treatment [14]. Due to the rarity of synovial sarcoma, clinical trials often encounter difficulties in patient recruitment, and novel therapies are relatively limited compared to other more common cancers. Many new approaches for the treatment of metastatic synovial sarcoma are currently under investigation, including targeted agents, epigenetic modulators, compounds that interfere with DNA damage response, and immunotherapy [14,15]. However, the impact of these strategies on improving synovial sarcoma outcomes remains limited [16]. Thus, long-term sustained preclinical and translational studies are still needed for this rare cancer.

Marine antimicrobial peptide TP4 was identified from Nile Tilapia (*Oreochromis niloticus*) and found to possess multiple biological functions [17,18], including anti-bacterial function [19,20], immunomodulation, and promotion of wound healing [20,21]. In addition, TP4 has received attention for its strong anticancer activity in glioblastoma cells [22], human non-small-cell lung cancer cells [23,24], and triple-negative breast cancer cells [25]. TP4 exerts its anticancer activity in different cancer cells through a variety of mechanisms. The FOSB signaling axis is the main signaling mechanism induced by TP4 in lung and breast cancer cells [25]. However, TP4 also induces mitochondrial dysfunction in glioblastoma cells [22]. In this study, we aimed to explore the anticancer activity of TP4 in human synovial sarcoma cells and to study the mechanisms underlying its anticancer effects.

## 2. Results

### 2.1. TP4 Decreases Cell Viability in Human Synovial Sarcoma Cells

**Figure 1:**
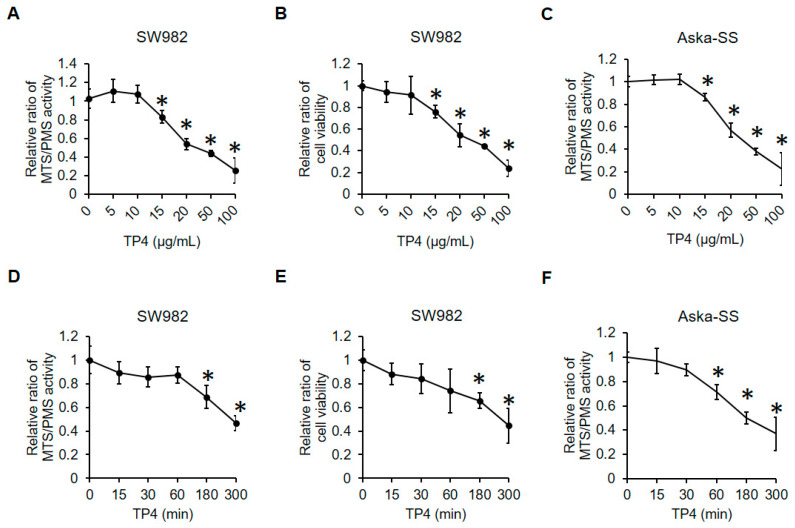
TP4 triggers cell death in synovial sarcoma cells. Dose-dependent cytotoxicity of TP4 in SW982 cells (**A**,**B**) and Aska-SS cells (**C**). Cells were incubated with various concentrations of TP4 for 5 h. Time-dependent cytotoxicity of TP4 in SW982 cells (**D**,**E**) and Aska-SS cells (**F**). Cells were treated with TP4 (20 μg/mL) for the indicated times. Cytotoxicity was determined by the MTS/PMS assay (**A**,**C**,**D**,**F**) and trypan blue exclusion assay (**B**,**E**). *: *p* < 0.05 (Compared to 0 min or 0 μg/mL). IC50 values were calculated with GraphPad Prism 8 using a non-linear regression fit (log(inhibitor) vs. normalized response—variable slope).

To examine the antisynovial sarcoma activity of TP4, human synovial sarcoma cells, SW982 and Aska-SS, were exposed to TP4 at doses ranging from 0 to 100 μg/mL. Five hours after exposure, cell viability was evaluated by MTS and trypan blue exclusion assays (Figure 1A–C). A dose-dependent cytotoxic effect was observed after exposure to TP4 in both SW982 and Aska-SS cells. TP4 doses below 10 μg/mL were not detrimental to either synovial sarcoma cell line. The half maximal inhibitory concentration (IC50) values from the MTS assay was calculated. The IC50 values were 29.20 and 32.41 μg/mL in SW982 and AsKa-SS cells, respectively. To study the time-course of cytotoxicity, cells were exposed to TP4 for different times (Figure 1D–F). Results showed that cytotoxic effects were observed after 1 h and 3 h after exposure to TP4 in SW982 cells and Aska-SS cells, respectively.

### 2.2. TP4 Induces Necrotic Cell Death in Human Synovial Sarcoma Cells

**Figure 2:**
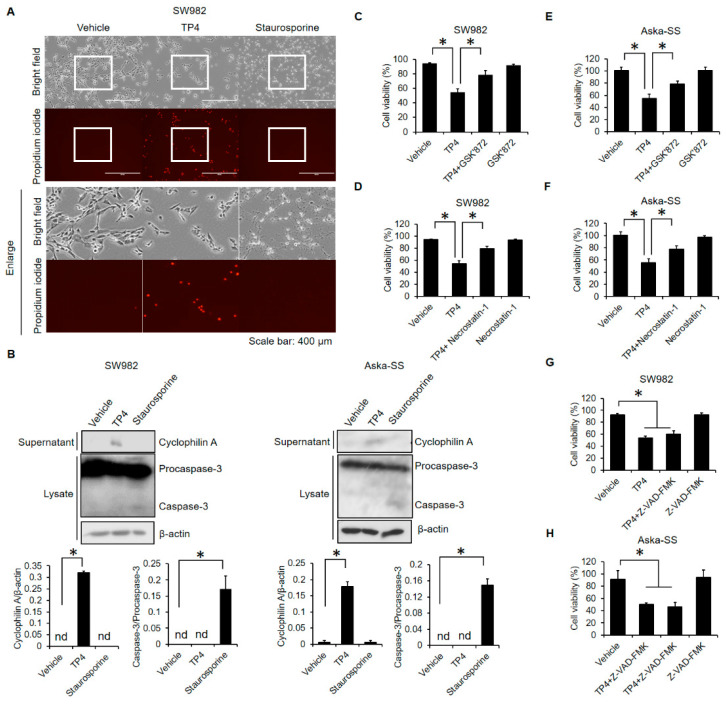
Necrotic cell death is induced by TP4 in synovial sarcoma cells. Cells were treated with TP4 (20 μg/mL) or staurosporine (1 μM) for 5 h and 24 h, respectively. Vehicle control was 0.5% DMSO. Necrotic cell death was determined by the propidium iodide incorporation assay (**A**). (**B**) Cells (SW982 and Aska-SS) were treated as described above. Cell lysates and culture supernatants were collected and immunoblotted with indicated antibodies. Band intensities were analyzed with ImageJ software. *: *p* < 0.05 (Compared to vehicle). Cells (SW982 and Aska-SS) were preincubated with GSK’872 (5 μM) (**C**,**E**), Necrostatin-1 (10 μM) (**D**,**F**), or Z-VAD-FMK (100 μM) (**G**,**H**) for 1 h, followed by TP4 (20 μg/mL) for additional 5 h. Cell viability was analyzed using the trypan blue exclusion assay. *: *p* < 0.05 (Vehicle vs. TP4; TP4 vs. TP4 + inhibitor). Vehicle control: appropriate amount of DMSO.

Necrotic cell death is the main cytotoxic pathway induced by TP4 in various cancer cell types [22,25], so we sought to test whether necrosis is also induced by TP4 in synovial sarcoma cells. Cells were treated with TP4 or the apoptotic inducing agent, stausporine, for 5 h, and necrotic cell death was analyzed with a propidium iodide (PI) incorporation assay. Cell shrinkage was observed after exposure to TP4 or stausporine; however, only exposure to TP4 increased the percentage of cells with incorporated PI (Figure 2A). Release of cyclophilin A into culture supernatant can serve as a marker for necrotic cell death [22,26], and we found that TP4 increased cyclophilin A in the supernatant, but not the apoptotic marker, caspase-3, in the lysate (Figure 2B). Furthermore, both GSK’872 and Necrostain-1 (necrotic inhibitors) effectively suppressed TP4-induced cytotoxicity in SW982 (Figure 2C,D) and Aska-SS cells (Figure 2E,F). In contrast, the apoptotic inhibitor, Z-VAD-FMK, failed to attenuate TP4-induced cell death in SW982 (Figure 2G) and Aska-SS cells (Figure 2H). These results strongly suggest that necrosis is the main type of cell death induced by TP4 in human synovial sarcoma cells.

### 2.3. TP4 Increased ROS Levels and Decreased Antioxidant Proteins

**Figure 3:**
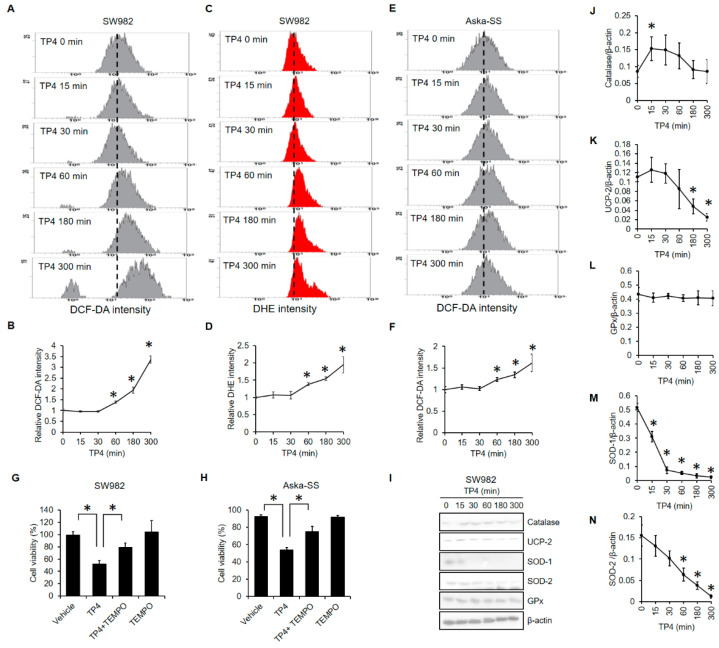
TP4 triggers oxidative stress. Synovial sarcoma cells were treated with TP4 (20 μg/mL) for the indicated times, and intracellular ROS was evaluated by DCF-DA (**A**,**B**,**E**,**F**) and DHE (**C**,**D**) fluorescence using flow cytometry. *: *p* < 0.05 (compared to 0 min). SW982 (**G**) and AsKa-SS (**H**) cells were preincubated with TEMPO (150 μM) for 1 h, followed by TP4 (20 μg/mL) for additional 5 h. Cell viability was determined using the trypan exclusion assay. *: *p* < 0.05 (Vehicle vs. TP4; TP4 vs. TP4 + TEMPO). (**I**) SW982 cells were treated with TP4 (20 μg/mL) for the indicated times, and cell lysates were collected and immunoblotted with the indicated antibodies. UCP-2: uncoupling protein-2; GPx: glutathione peroxidase; SOD-1: superoxide dismutase-1; SOD-2: superoxide dismutase-2. (**J**–**N**) Band intensities were analyzed with ImageJ. *: *p* < 0.05 (Compared to 0 min).

Excess ROS is often involved in necrotic cell death [27]. To test the role of ROS in TP4-induced cell death, we tracked ROS levels on a time-course after TP4 treatment of SW982 and AsKa-SS cells. Increased ROS levels were detectable 60 min after TP4 exposure in both SW982 (Figure 3A–D) and AsKa-SS cells (Figure 3E,F). Importantly, pretreatment with the ROS scavenger, TEMPO, blocked TP4-induced cell death in both SW982 (Figure 3G) and AsKa-SS cells (Figure 3H), indicating an essential role for ROS in TP4-induced cell death. Furthermore, we measured the protein levels of several antioxidant proteins, including catalase, uncoupling protein (UCP)-2, glutathione peroxidase, superoxide dismutase (SOD)-1, and SOD-2, in SW982 cells after TP4 exposure (Figure 3I–N). We found that TP4 dramatically reduced the levels of UCP-2, SOD-1, and SOD-2.

### 2.4. TP4 Initiates Mitochondrial Hyperpolarization

**Figure 4:**
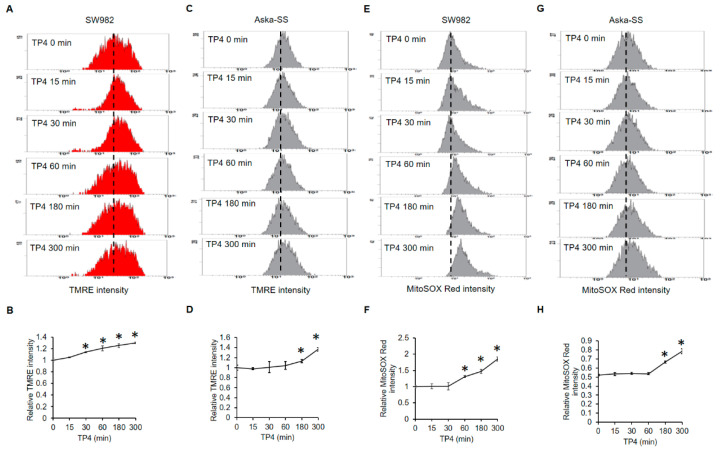
TP4 induces mitochondrial hyperpolarization and mitochondrial ROS generation. SW982 (**A**,**B**) and AsKa-SS (**C**,**D**) cells were treated with TP4 (20 μg/mL) for 0, 15, 30, 60, 180 and 300 min. TMRE intensity was analyzed by flow cytometry. SW982 (**E**,**F**) and AsKa-SS (**G**,**H**) cells were treated with TP4 (20 μg/mL), as described in (**A**–**D**), followed by MitoSOX Red (5 μM) for an additional 15 min. MitoSOX Red fluorescence intensity was determined by flow cytometry. *: *p* < 0.05 (Compared to 0 min).

Excessive ROS are often generated by damaged mitochondria [28]. Therefore, we next checked whether TP4 impairs mitochondrial function. Mitochondrial function was evaluated with the mitochondrial membrane potential indicating dye, TMRE. TP4 elevated TMRE intensities in both SW982 and AsKa-SS cell lines at different time-points. In SW982 cells, TMRE intensity was significantly induced after 30 min of TP4 exposure (Figure 4A,B). In AsKa-SS cells, TMRE intensity was elevated at 180 and 300 min after treatment (Figure 4C,D). These results indicated that TP4 induces mitochondrial hyperpolarization. Thus, we further assessed whether mitochondrial ROS were increased by TP4. Mitochondrial ROS was evaluated by a specific mitochondrial ROS indicator, MitoSOX Red. We found that TP4 increased MitoSOX Red intensity in SW982 cells at 60, 180 and 300 min after treatment (Figure 4E,F). In addition, MitoSOX Red intensity was elevated by TP4 in AsKa-SS cells at 180 and 300 min after TP4 treatment (Figure 4G,H). Together, the data showed that TP4 induced mitochondrial hyperpolarization, which was coincident with excessive mitochondrial ROS production.

### 2.5. Calcium-Overload Is Required for TP4-Induced Cytotoxicity

**Figure 5:**
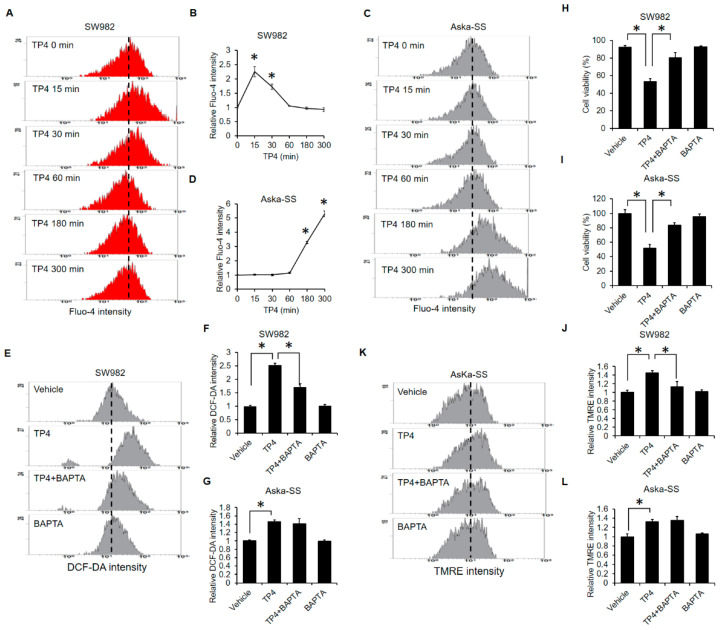
TP4 triggers calcium overload. SW982 (**A**,**B**) and AsKa-SS (**C**,**D**) cells were preloaded with Fluo-4 (5 μM) for 15 min, followed by TP4 treatment. After 0, 15, 30, 60, 180 or 300 min, Fluo-4 intensity was analyzed by flow cytometry. *: *p* < 0.05 (compared to 0 min). SW982 (**E**,**F**) and Aska-SS (**G**) cells were preincubated with BAPTA (10 μM) for 1 h, followed by TP4 (20 μg/mL) for 5 h. Then, cells were incubated with DCF-DA for 15 min. DCF-DA intensity was analyzed by flow cytometry. *: *p* < 0.05 (Vehicle vs. TP4; TP4 vs. TP4 + BAPTA). SW982 (**H**) and AsKa-SS (**I**) cells were preincubated with BAPTA (10 μM) for 1 h, followed by TP4 (20 μg/mL) for an additional 5 h. Cell viability was determined using the trypan exclusion assay. *: *p* < 0.05 (Vehicle vs. TP4; TP4 vs. TP4 + BAPTA). SW982 (**J**) and AsKa-SS (**K**,**L**) cells were incubated with TP4 (20 μg/mL) for 300 min. TMRE intensity was analyzed by flow cytometry. *: *p* < 0.05 (Vehicle vs. TP4; TP4 vs. TP4 + BAPTA).

Similar to ROS, calcium plays an important role in necrotic cell death [29]. Thus, we assessed whether TP4 modulates intracellular calcium levels. We found that TP4 elevates intracellular calcium levels in both SW982 (Figure 5A,B) and AsKa-SS (Figure 5C,D) cells. However, TP4-induced elevation of intracellular calcium was more rapid and transient in SW982 cells than it was in AsKa-SS cells. The calcium chelator, BAPTA, abolished TP4-induced ROS generation in SW982 cells (Figure 5E,F) but not in AsKa-SS cells (Figure 5G,H). These results suggested that calcium acts upstream of ROS during TP4-induced necrotic cell death in SW982 cells. In contrast, ROS production was independent of calcium in AsKa-SS cells. Nevertheless, BAPTA abolished TP4-induced cell death in both SW982 (Figure 5H) and AsKa-SS (Figure 5I) cells, suggesting that calcium is required for TP4-induced cytotoxicity in both lines. Notably, BAPTA attenuated TP4-induced mitochondrial hyperpolarization only in SW982 cells (Figure 5J) and not in AsKa-SS cells (Figure 5K,L). Thus, calcium overload appears to induce mitochondrial damage in TP4-treated SW982 cells, while the TP4-induced calcium overload may occur simultaneously with or after mitochondrial damage in AsKa-SS cells. Taken together, our experiments suggest that calcium overload, excessive ROS production and mitochondrial hyperpolarization are all involved in TP4-induced cytotoxicity in both SW982 and AsKa-SS cells, but the induction patterns and interdependencies of these events are different in the two synovial sarcoma cell lines.

## 3. Discussion

In a previous study on triple-negative breast cancer cells, TP4 was shown to induce FOSB activation via elevation of intracellular calcium [25]. Here, we found that TP4 also disrupts calcium homeostasis in synovial sarcoma cells (Figure 5A–D); however, the activation status of FOSB in TP4-treated synovial sarcoma cells remains unclear. Furthermore, the circumstances of TP4-induced intracellular calcium elevation appear to differ in SW982 and AsKa-SS cells (Figure 5A–D). Although the calcium chealtor, BAPTA, abolished TP4-induced cell death in both SW982 and AsKa-SS cells (Figure 5H,I), the different calcium-induction timings suggest that TP4 might induce different signaling axes in these two synovial sarcoma cell lines. In AsKa-SS cells, BAPTA failed to block TP4-induced ROS generation (Figure 5G), which suggests ROS is upstream or independent of calcium overload. The timelines of TP4-induced ROS (Figure 3E,F) and calcium (Figure 5C,D) in AsKa-SS cells also support this inference. Elevated ROS was observed at 60 min after TP4 exposure (Figure 3E,F), while TP4-induced elevation of intracellular calcium was observed later, at 180 min (Figure 5C,D).

Excessive ROS is known to damage intracellular biological molecules such as nucleic acids, proteins, and lipids, which leads to cell death through various mechanisms [30]. Therefore, modulation of intracellular ROS levels may be a valuable strategy for cancer treatments [30]. Many clinically approved anticancer drugs induce cell death through mechanisms that involve increased levels of ROS; examples include: anthracyclin/doxorubicin, 5-fluorouracil, Erlotinib, and platinum-based drugs, among others [30]. Furthermore, cancer cells may acquire resistance to these anticancer drugs by elevating levels of endogenous free radical scavengers [31]. For example, SOD2 activity is a determinant of radiation therapy efficacy in murine sarcoma [32,33]. Additionally, elevated levels of GPx contribute to doxorubicin resistance in a human sarcoma cell line [34], and high glutathione level is positively correlated with metastatic characteristics and disease stage in human soft tissue sarcoma [35]. In contrast, bone and soft tissue sarcoma patients exhibit reduced levels of catalase and oxidative damage, which may be associated with genetic instability and early-stage tumor development [36].

We found that TP4 induces mitochondrial hyperpolarization and subsequent elevation of mitochondrial ROS (Figure 4). Moreover, TP4 downregulates the levels of UCP-2, SOD1, and SOD2 (Figure 4), which would be expected to increase cell vulnerability to ROS-mediated damage. Notably, high levels of SOD2 and GPx contribute to therapeutic resistance in soft tissue sarcoma [32,33,34]. Therefore, TP4 may be able to enhance the efficacy of current synovial sarcoma therapeutics, due to its downregulation of antioxidant proteins. In addition to its antioxidant activity, UCP-2 regulates ATP production and the proton gradient within mitochondria [37], and UCP-2 knockout cells exhibit elevated mitochondrial membrane potential [38]. Correspondingly, we found that TP4-induced mitochondrial hyperpolarization (Figure 4A,B) is accompanied by reduced UCP-2 level (Figure 3I) and increased mitochondrial ROS generation (Figure 4E,F).

TP4 was previously found to preferentially bind to mitochondria and the mitochondrial membrane protein, adenine nucleotide translocase 2, in non-small cell lung cancer cells and triple-negative breast cancer cells [25,39]. In the present study, we demonstrated that TP4 induces mitochondrial hyperpolarization in synovial sarcoma cells. Thus, the mitochondria seem to be a major intracellular target of TP4 in different types of cancer cells.

TP4 is very different from conventional chemotherapeutic agents. While current chemotherapeutic agents mainly induce apoptotic cell death [40], TP4 induces necrotic cell death (Figure 2). This mode of death might be an advantage for cancer treatments. Apoptosis is a reversible process before cells reach the point-of-no-return, nuclear fragmentation [41], and this reversibility might benefit cancer cells by allowing them to develop chemoresistance. In contrast, necrosis is an irreversible cell death process [42], which might make chemoresistance less likely.

In this study, we successfully demonstrated that TP4 exhibits strong anticancer activity against synovial sarcoma cells in vitro. Furthermore, we found that in this context, TP4 induces calcium overload, mitochondrial dysfunction, ROS production, and impaired antioxidant defense, followed by necrotic cell death. Further studies are required to determine whether TP4 can also cause synovial sarcoma cell death in vivo.

## 4. Materials and Methods

### 4.1. Reagents

Phenazine methosulfate (PMS) and 3-(4,5-dimethylthiazol-2-yl)-5-(3-carboxymethoxyphenyl)-2-(4-sulfophenyl)-2H-tetrazolium inner salt (MTS) were purchased from Promega (Madison, WI, USA). GSK’872 was purchased from BioVision (Milpitas, CA, USA). Z-VAD-FMK was purchased from Cell Signaling Technology (Danvers, MA, USA). DMSO, trypan blue, propidium iodide (PI), Necrostatin-1, stausporine, dihydroethidium (DHE), 2′,7′-dichlorodihydrofluorescein diacetate (DCFDA), TEMPO and BAPTA were purchased from Sigma-Aldrich (Merck KGaA, Darmstadt, Germany). Tetramethylrhodamine, Ethyl Ester, Perchlorate (TMRE), MitoSOX Red and Fluo-4, AM (Fluo-4) were purchased from ThermoFisher (Waltham, MA, USA). Tilapia piscidin 4 (TP4; H-FIHHIIGGLFSAGKAIHRLIRRRRR-OH) peptide was synthesized by GL Biochem (Shanghai, China).

### 4.2. Cell Culture and Treatments

The SW982 human synovial sarcoma cell line was purchased from ATCC (The Global Bioresource Center; Manassas, VA, USA). The Aska-SS human synovial sarcoma cell line was purchased from RIKEN BioResource Research Center (Koyadai, Ibaraki, Japan). SW982 cells were cultured as described previously [26]. Aska-SS cells were maintained in Dulbecco’s Modified Eagle’s medium (DMEM; ThermoFisher), supplemented with 20% fetal bovine serum (Biological Industries; Cromwell, CT, USA) and penicillin-streptomycin (Biological Industries; Cromwell, CT, USA). Cells were treated as described in the figure legends.

### 4.3. Cytotoxicity and PI Exclusion Assay

Cytotoxicity was analyzed by the MTS/PMS and trypan exclusion assays. Trypan blue exclusion was performed as described previously [22]. For the MTS/PMS assay, cells were incubated with MTS/PMS mixture according to the manufacturer’s instructions for an additional 0.5 h. Absorbance at 490 nm was recorded using microplate reader. PI exclusion assay was performed as described previously [22].

### 4.4. Western Blot

After treatment, cell lysates and culture supernatants were collected. Cell lysates were collected for detection of intracellular proteins (caspase-3, catalase, UCP-2, SOD-1, SOD-2, GPx and β-actin). Supernatants were collected for detection of cyclophilin A, according to a previously described procedure [22]. Antibodies were purchased from Cell Signaling Technology (Danvers, MA, USA). Cell lysates and supernatants were separated, transferred and immunoblotted with indicated antibodies. Band intensity was quantified with ImageJ software 64-bit Java 1.8.0_172 (NIH, Bethesda, MA, USA).

### 4.5. Flow Cytometry

For DHE, cells were preloaded with DHE (20 μM) for 1 h, followed by TP4 (20 μg/mL) treatment. DHE intensity was measured by flow cytometry (Beckman Coulter, Indianapolis, IN, USA). For Fluo-4, cells were preloaded with Fluo-4 (5 μM) for 15 min, followed by TP4 (20 μg/mL) treatment. Fluo-4 intensity was measured by flow cytometry (Beckman Coulter, Indianapolis, IN, USA). For DCF-DA, TMRE and MitoSOX Red, cells were incubated with TP4 for indicated times, followed by DCF-DA (10 μM), TMRE (100 nM) or MitoSOX Red (5 μM) for 15 min. Fluorescence intensities of DCF-DA, TMRE and MitoSOX Red were measured by flow cytometry (Beckman Coulter, Indianapolis, IN, USA).

### 4.6. Statistical Analysis

All experiments were performed in triplicate with at least three replicates. Data are represented as mean ± SEM. Student’s *t*-test and one-way ANOVA were used as appropriate for data analyses. *p* < 0.05 was considered statistically significant.

## 5. Conclusions

In the present study, our in vitro data suggest potential therapeutic benefits of the marine antimicrobial peptide, TP4, in human synovial sarcoma. TP4 causes necrosis in synovial sarcoma cells via induction of calcium overload, mitochondrial hyperpolarization, and oxidative stress.

## Data Availability

The data presented in this study are available on request from the corresponding authors.
